# Correction: Publicly available data reveals association between asthma hospitalizations and unconventional natural gas development in Pennsylvania

**DOI:** 10.1371/journal.pone.0309010

**Published:** 2024-08-13

**Authors:** Anna Bushong, Thomas McKeon, Mary Regina Boland, Jeffrey Field

Table 2 is a duplicate of Table 1. Please view the correct Table 2 below.

**Table 2:** Results of the multiple linear regression model using only rural counties to investigate the relationship between UNGD and asthma hospitalization.

Model 2	Range	Unit Increase	Associated % Change in Asthma HAR	95% Confidence Intervals (as % Change)	p-value (α = 0.05)
**Annual well density**	0.00–0.43 wells/sq mi	0.01 wells/sq mi	+3.31%	[1.14%, 7.70%]	3.76 x 10^−5^
**Median Income**	$34,018 - $60,068	$1000	-1.90%	[-2.64%, -1.16%]	4.77 x 10^−7^
**% White Population**	71–99%	1%	-2.54%	[-3.72%, -1.35%]	3.03 x 10^−5^
**PM**_**2.5**_	7.8–16.8 μg/m^3^	1 μg/m^3^	+7.33%	[4.13%, 10.62%]	4.02 x 10^−6^

Ranges for variables are based on counties included in the model. Since the response variable was natural log-transformed, the associated percent change in asthma HAR for each explanatory variable was computed from through backtransforming the partial slopes of the regression model. The 95% confidence intervals were computed using the raw partial slope and standard error, and then backtransforming the lower and upper bounds. A percent change highlighted in green represents an associated percent increase, while highlight in red represents an associated percent decrease.
