# Supplementary material for: Publicly available data reveals association between asthma hospitalizations and unconventional natural gas development in Pennsylvania
Source: PLoS One. 2022 Mar 31;17(3):e0265513. doi: 10.1371/journal.pone.0265513 (PMC8970380; doi:10.1371/journal.pone.0265513)

S1 Figure: Line graphs comparing trends of asthma HAR and average annual PM 2.5 concentrations in rural and urban counties.
Panel A shows a line graph for rural counties with average asthma HAR (per 10,000 cases) on the left axis in purple and average annual PM 2.5 concentrations (μg/m^3) on the right axis in gray over time. Panel B shows a line graph for urban counties of average asthma HAR (per 10,000 cases) on the left axis in purple and average annual PM 2.5 concentrations (μg/m^3) on the right axis in gray over time.

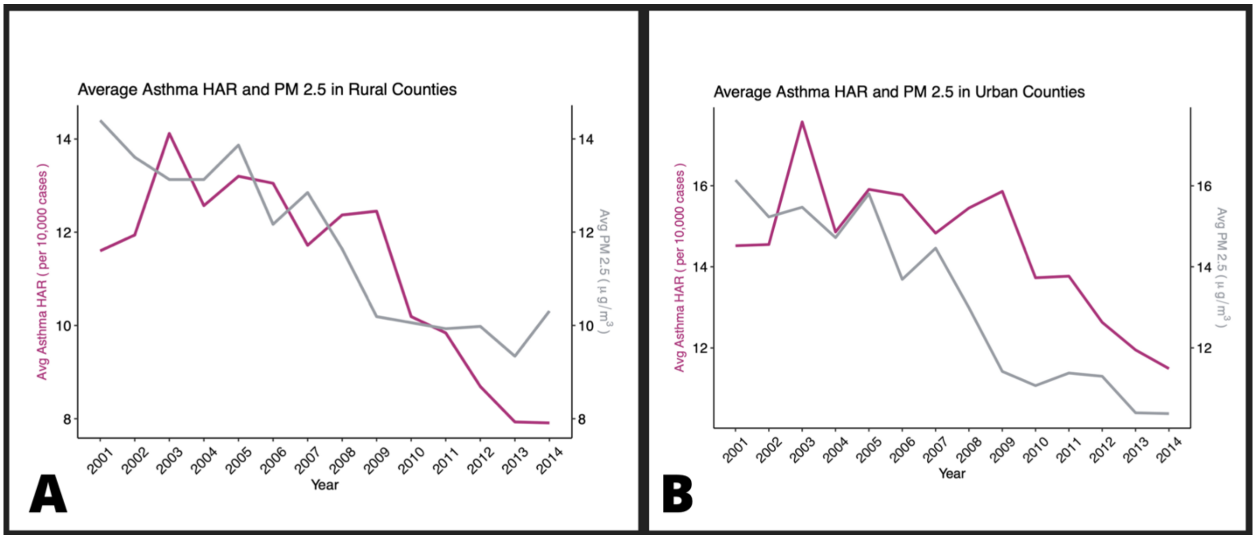

Supplement: S1 Fig — Panel A shows a line graph for rural counties with average asthma HAR (per 10,000 cases) on the left axis in purple and average annual PM 2.5 concentrations (μg/m3) on the right axis in gray over time. Panel B shows a line graph for urban counties of average asthma HAR (per 10,000 cases) on the left axis in purple and average annual PM 2.5 concentrations (μg/m3) on the right axis in gray over time. (PDF) [file pone.0265513.s001.pdf]
