# Supplementary material for: Publicly available data reveals association between asthma hospitalizations and unconventional natural gas development in Pennsylvania
Source: PLoS One. 2022 Mar 31;17(3):e0265513. doi: 10.1371/journal.pone.0265513 (PMC8970380; doi:10.1371/journal.pone.0265513)
Supplement: S1 Table — Ranges for PM 2.5 are based on counties included in the model. Since the response variable was natural log-transformed, the associated percent change in asthma HAR for the explanatory variable, PM 2.5, was computed from through backtransforming the partial slope. A percent change highlighted in green represents an associated percent increase. (PDF) [file pone.0265513.s002.pdf]

S1 Table: Results from a model using rural PA counties: relationship between asthma HAR and average annual PM 2.5.
Ranges for PM 2.5 are based on counties included in the model.
Since the response variable was natural log-transformed, the associated percent change in asthma HAR for the explanatory variable, PM 2.5, was computed from through backtransforming the partial slope. A percent change highlighted in green represents an associated percent increase.

	**Range**	**Unit Increase**	**Associated % Change in Asthma HAR**	**95% Confidence Interval (as % Change)**	**p-value** ($\alpha = 0.05$)
**PM $_{2.5}$**	7.8 - 16.8 $\mu g/m^3$	1 $\mu g/m^3$	+7.52%	[4.16%, 10.97%]	6.30×10^{-6}
