# Supplementary material for: Publicly available data reveals association between asthma hospitalizations and unconventional natural gas development in Pennsylvania
Source: PLoS One. 2022 Mar 31;17(3):e0265513. doi: 10.1371/journal.pone.0265513 (PMC8970380; doi:10.1371/journal.pone.0265513)
Supplement: S3 Table — Cells highlighted in blue indicate counties that did not have data available for age-adjusted asthma HAR for the years of study. Cells highlighted in yellow indicate counties with incomplete data available for age-adjusted asthma HAR for the years of study. Cells with * indicates a county in the third quartile for HF well count based on the cumulative well count in 2014. Cells with ** indicates a county in the fourth quartile for HF well count based on the cumulative well count in 2014. (PDF) [file pone.0265513.s004.pdf]

**S3 Table:** List of the Pennsylvanian counties included in each model with information on age-adjusted asthma HAR data available and prevalence of HF wells.

Cells highlighted in blue indicate counties that did not have data available for age-adjusted asthma HAR for the years of study. Cells highlighted in yellow indicate counties with incomplete data available for age-adjusted asthma HAR for the years of study. Cells with * indicates a county in the third quartile for HF well count based on the cumulative well count in 2014. Cells with ** indicates a county in the fourth quartile for HF well count based on the cumulative well count in 2014.

| Model 1 (Statewide, n=67) | Model 2 (Rural only, n=48) | Model 3 (Geisinger, n=30) |
|---|---|---|
| Adams | Adams | Blair |
| Allegheny* | Armstrong* | Bradford** |
| Armstrong* | Bedford | Cambria |
| Beaver | Blair | Carbon |
| Bedford | Bradford** | Centre |
| Berks | Butler** | Clearfield* |
| Blair | Cambria | Columbia |
| Bradford** | Carbon | Elk* |
| Bucks | Centre | Huntingdon |
| Butler** | Clearfield* | Jefferson |
| Cambria | Columbia | Lycoming** |
| Carbon | Crawford | McKean* |
| Centre | Elk* | Mifflin |
| Chester | Fayette** | Monroe |
| Clearfield* | Franklin | Northumberland |
| Columbia | Greene** | Schuylkill |
| Crawford | Huntingdon | Susquehanna** |
| Cumberland | Indiana | Wayne |
| Dauphin | Jefferson | Wyoming* |
| Delaware | Lawrence | Cameron |
| Elk* | Lycoming** | Clinton* |
| Erie | McKean* | Juniata |
| Fayette** | Mercer | Montour |
| Franklin | Mifflin | Perry |
| Greene** | Monroe | Pike |
| Huntingdon | Northumberland | Potter |
| Indiana | Schuylkill | Snyder |
| Jefferson | Somerset | Sullivan* |
| Lackawanna | Susquehanna** | Tioga** |
| Lancaster | Venango | Union |

| | | |
|---|---|---|
| Lawrence | Washington** | |
| Lebanon | Wayne | |
| Lehigh | Wyoming* | |
| Luzerne | Cameron | |
| Lycoming** | Clarion | |
| McKean* | Clinton* | |
| Mercer | Forest | |
| Mifflin | Fulton | |
| Monroe | Juniata | |
| Montgomery | Montour | |
| Northampton | Perry | |
| Northumberland | Pike | |
| Philadelphia | Potter | |
| Schuylkill | Snyder | |
| Somerset | Sullivan* | |
| Susquehanna** | Tioga** | |
| Venango | Union | |
| Washington** | Warren | |
| Wayne | | |
| Westmoreland* | | |
| Wyoming* | | |
| York | | |
| Cameron | | |
| Clarion | | |
| Clinton* | | |
| Forest | | |
| Fulton | | |
| Juniata | | |
| Montour | | |
| Perry | | |
| Pike | | |
| Potter | | |
| Snyder | | |
| Sullivan* | | |
| Tioga** | | |
| Union | | |
| Warren | | |

Note: Tioga only lacks age-adjusted asthma HAR for 2010.
